# U-Shaped relationship of insulin-like growth factor I and incidence of nonalcoholic fatty liver in patients with pituitary neuroendocrine tumors: a cohort study

**DOI:** 10.3389/fendo.2024.1290007

**Published:** 2024-02-02

**Authors:** Yan Hu, Chen Yuan, Muila Abdulnaimu, Jimilanmu Memetmin, Zhang Jie, Aihemaitijiang Tuhuti, Hanikzi Abudueini, Yanying Guo

**Affiliations:** ^1^ Graduate School, Xinjiang Medical University, Urumqi, China; ^2^ Department of Endocrinology, People’s Hospital of Xinjiang Uygur Autonomous Region, Xinjiang Clinical Research Center for Diabetes, Urumqi, China

**Keywords:** U-shaped, insulin-like growth factor I, nonalcoholic fatty liver disease, pituitary neuroendocrine tumors, cohort study

## Abstract

**Context:**

Although the role of insulin-like growth factor I (IGF-1) in nonalcoholic fatty liver disease (NAFLD) has garnered attention in recent years, few studies have examined both reduced and elevated levels of IGF-1.

**Objective:**

The aim of this study was to examine the potential relationship between IGF-1 levels and the risk of new-onset NAFLD in patients with pituitary neuroendocrine tumors (PitNET).

**Methods:**

We employed multivariable Cox regression models and two-piecewise regression models to assess the association between IGF-1 and new-onset NAFLD. Hazard ratios (HRs) and their corresponding 95% confidence intervals (CIs) were calculated to quantify this association. Furthermore, a dose-response correlation between lgIGF-1 and the development of NAFLD was plotted. Additionally, we also performed subgroup analysis and a series sensitivity analysis.

**Results:**

A total of 3,291 PitNET patients were enrolled in the present study, and the median duration of follow-up was 65 months. Patients with either reduced or elevated levels of IGF-1 at baseline were found to be at a higher risk of NAFLD compared to PitNET patients with normal IGF-1(log-rank test, P < 0.001). In the adjusted Cox regression analysis model (model IV), compared with participants with normal IGF-1, the HRs of those with elevated and reduced IGF-1 were 2.33 (95% CI 1.75, 3.11) and 2.2 (95% CI 1.78, 2.7). Furthermore, in non-adjusted or adjusted models, our study revealed a U-shaped relationship between lgIGF-1 and the risk of NAFLD. Moreover, the results from subgroup and sensitivity analyses were consistent with the main results.

**Conclusions:**

There was a U-shaped trend between IGF-1 and new-onset NAFLD in patients with PitNET. Further evaluation of our discoveries is warranted.

## 1 Introduction

Nonalcoholic fatty liver disease (NAFLD) has emerged as a leading cause of chronic liver disease globally and is likely to be a major contributor to end-stage liver failure in the future (1–3). NAFLD is a complex liver disease characterized by steatosis, hepatic inflammation, fibrosis (4–6) and is closely associated with other metabolic disorders (7, 8). In addition, individuals with NAFLD are more likely to experience all-cause mortality, with cardiovascular events being the primary cause among those affected (9, 10).

Pituitary neuroendocrine tumors (PitNET) include functional tumors that autonomously secrete pituitary hormones and nonfunctional tumors that are not associated with excess hormones (11, 12). Growth hormone (GH)-secreting pituitary adenomas, marked by elevated levels of GH and IGF-1, account for approximately 12% of functional pituitary tumors (13). Persistently increased secretion of GH and IGF-1 can result in liver hypertrophy, which is a form of visceral hypertrophy, and can disrupt glucose and lipid metabolism, leading to multiple comorbidities (12). A meta-analysis has reported a 2-fold increase in all-cause mortality among patients with functional GH cell adenomas (14). However, one recent study has shown that patients who receive appropriate treatment and maintain normal serum IGF-1 levels are not at increased risk of death (15).

PitNET can also cause hypopituitarism due to its occupying effect (16), and the deficiency of pituitary hormones usually begins with the GH/IGF-1 axis (17). However, GH/IGF-1 deficiency is often not adequately addressed in hormone replacement therapy (18). Indeed, several reports have defined a significant connection between NAFLD and the GH/IGF-1 axis (4, 19). Patients with NAFLD have reduced levels of IGF-1 (20), and IGF-1 deficiency can facilitate the development and advancement of NAFLD (21). In adolescents and adults with NAFLD patients, recombinant human growth hormone (rhGH) supplementation therapy has been shown to significantly improve hepatic steatosis and fibrosis (22, 23). Additionally, IGF-1 has demonstrated anti-fibrotic properties in rodent models of NAFLD (24).

GH-secreting pituitary adenomas can result in overproduction of IGF-1, while other types of PitNET can lead to IGF-1 deficiency due to their occupying effect. This makes individuals with PitNET an ideal group to study the connection between IGF-1 levels and NAFLD. Generally, reduced IGF-1 levels are associated with NAFLD. However, there may also be positive connections between elevated IGF-1 and NAFLD, indicating a potential nonlinear relationship between IGF-1 levels and NAFLD occurrence. Consequently, we hypothesize that in individuals with PitNET, both elevated and reduced levels of IGF-1 are linked to an increased risk of developing NAFLD.

## 2 Materials and methods

### 2.1 Study population

**Figure 1:**
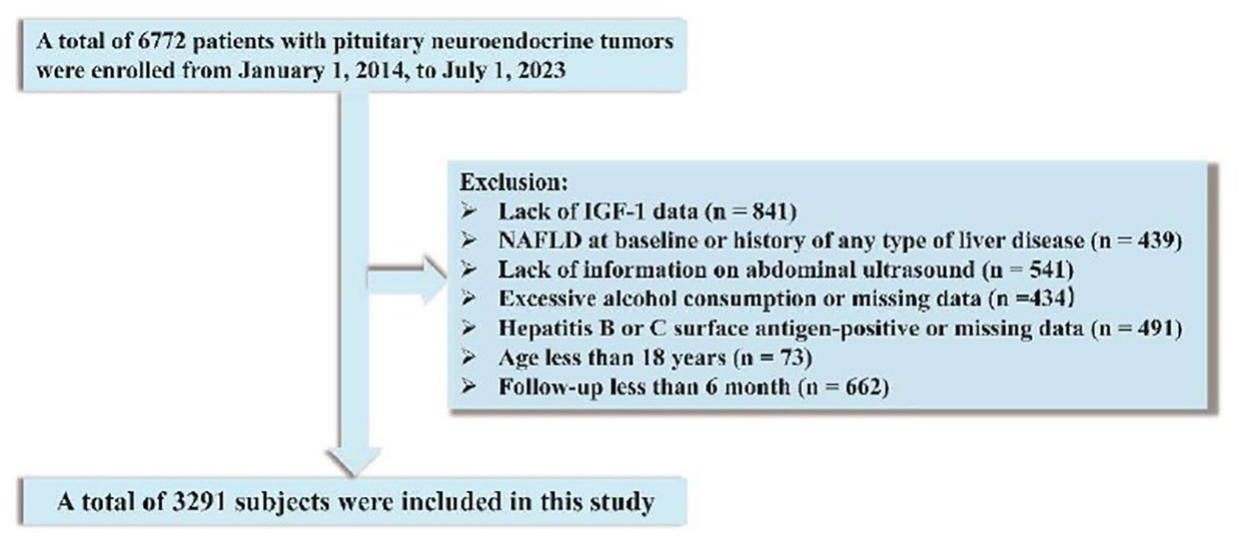
Flowchart of the participant selection process.

We recruited a total of 6772 patients diagnosed with PitNET in the period from January 1, 2014, to July 1, 2023, at the People’s Hospital of Xinjiang Uygur Autonomous Region. Excluded were patients with no available data on IGF-1 (n = 841), preexisting NAFLD or any type of liver disease (n = 439), lack of information on abdominal ultrasound (n = 541), excessive alcohol consumption or unavailability of data on alcohol consumption (n = 434), hepatitis B or C antigen-positive status or missing data (n = 491), age less than 18 years (n = 73), and follow-up time less than 6 month (n = 662). Ultimately, the final analysis included 3,291 patients after these exclusions (**Figure 1**). Approval was received from the Ethical Committee of the People’s Hospital of Xinjiang Uygur Autonomous Region (No.KY2023013149). The need for written informed consent was waived owing to the retrospective nature of the study. We adhered to the Strengthening the Reporting of Observational Studies in Epidemiology (STROBE) guideline recommendations for reporting our findings (25).

### 2.2 Data collection

#### 2.2.1 Clinical variables collection

Information on demographics, lifestyle behaviors, and medical history was extracted from the electronic medical records of all study participants. Weight, height, and blood pressure were measured using standard protocols. Details regarding variable definitions are provided in the **Supplementary Materials**. Medical history was assessed according to International Classification of Diseases, 10th Revision (ICD-10) codes. Medical history included diabetes, hypertension, hyperlipemia, NAFLD, and any type of liver disease.

#### 2.2.2 Lab testing

Participants were fasting for at least eight hours before the blood draw. Fasting blood glucose (FBG), low-density lipoprotein cholesterol (LDL-C), high-density lipoprotein cholesterol (HDL-C), total cholesterol (TC), triglycerides (TG), uric acid (UA), and hypersensitive C-reactive protein (hs-CRP) were measured in the central laboratory of the People’s Hospital of Xinjiang Uygur Autonomous Region. IGF-1 was quantified using the DPC Immulite 2000 chemiluminescence analyzer (Siemens AG, Munich, Germany) by the immunochemiluminescence method. Serum prolactin is measured by immunometric assays. Adrenal insufficiency had to be established through appropriate stimulation tests (26). Assessment of additional function of the pituitary using baseline measurements of free triiodothyronine, free thyroxine, thyroid-stimulating hormone (TSH), follicle-stimulating hormone (FSH), luteinizing hormone (LH), and testosterone (in males) or estradiol (in females). Details of pituitary function tests are provided in the **Supplementary Materials**.

### 2.3 Definition

IGF-1 at baseline was categorized into three groups based on age- and sex-adjusted reference ranges. Reduced IGF-1, namely, IGF-1<-2 standard deviation score (SDS) for sex and age (27). Elevated IGF-1, namely, IGF-1>2SDS for sex and age.

### 2.4 Follow-up and outcome assessment

Participants were assessed for NAFLD by abdominal ultrasound once a year during follow-up. NAFLD was diagnosed based on the diagnostic criteria established previously (28–30). The diagnostic criteria for NAFLD are provided in the **Supplementary Materials**.

The follow-up period (measured in months) of each participant was calculated from the baseline date to the earliest of the subsequent dates: occurrence of NAFLD, death, loss to follow-up, or July 1, 2023 (the end of the study’s follow-up), for patients who did not have an endpoint event during follow-up but had their last clinical visit. The outcome was the first occurrence of NAFLD. These outcome events were reviewed and adjudicated centrally by an independent clinical events committee.

### 2.5 Statistical analysis

IGF-1 at baseline was categorized into three groups according to age- and sex- adjusted reference ranges: normal IGF-1, elevated IGF-1, and reduced IGF-1. The cumulative incidence of NAFLD across different IGF-1 categories was assessed and compared by using Kaplan-Meier plots and log-rank tests. We evaluated collinearity in all models by measuring the variance inflation factor (VIF) and using a pre-set threshold of 5 as an indication of multicollinearity (**Supplementary Table 1**). Any variables with a VIF above 5 were excluded. Hazard ratios (HRs) and 95% confidence intervals (CIs) for NAFLD were calculated by univariable and multivariable models with Cox regression analysis. IGF-1 was assessed as a categorical and continuous variable. Given a non-normal distribution, IGF-1 was lg_10_-transformed for the continuous model. The dose-response relationship between lgIGF-1 and the risk of developing NAFLD was plotted in non-adjusted and adjusted models. Concerning the nonlinear relationship, we utilized a two-piecewise linear regression model to investigate the threshold effect of lgIGF-1 on the occurrence of NAFLD, employing a smoothing function. Moreover, we performed subgroup analyses and reported interactions. In addition, sensitivity analyses were performed to ensure the robustness of our findings. Detailed information regarding the statistical analysis can be found in the **Supplementary Materials**. Statistical tests were performed using R version 4.1.1, and P < 0.05 (two-sided) was considered statistically significant.

## 3 Results

### 3.1 Characteristics of study participants

Baseline characteristics of the participants grouped by IGF-1 are shown in **Table 1**. We divided IGF-1 into three groups according to age- and sex-adjusted reference ranges. Totally, 3291 subjects were enrolled in this cohort study, of whom 2122 (64.5%) were classified as normal IGF-1, 214(6.5%) were classified as elevated IGF-1, and 955(29%) were classified as reduced IGF-1. The mean age of the participants was 45.08 ± 14.05 years, with females accounting for 66%. Compared with patients with normal IGF-1, those with elevated IGF-1 and reduced IGF-1 were more likely to have a higher BMI, UA, TG, FBG, number of pituitary deficiencies, and lower HDL-C. They were also more likely to have hypertension, diabetes, hyperlipemia, diabetes insipidus, aggressive tumors, pituitary insufficiency, gonad axis insufficiency, thyroid axis insufficiency, adrenal axis insufficiency, and a giant adenoma at baseline.

**Table 1:** Baseline characteristics of the study participants according to IGF-1 ranked variable.

Variables	Normal IGF-1	Elevated IGF-1	Reduced IGF-1	P-value
No. of participants	2122	214	955	
Age (years)	44.59 ± 14.59	45.13 ± 11.97	46.14 ± 13.18	0.001
PRL (ng/ml)	26.31 (12.76-51.58)	13.32 (8.57-28.56)	22.00 (12.00-46.15)	<0.001
BMI (kg/m²)	25.02 ± 4.11	26.15 ± 4.13	27.81 ± 5.16	<0.001
UA (umol/L)	282.76 ± 94.64	268.52 ± 86.37	314.17 ± 96.52	<0.001
TC (mmol/L)	4.37 ± 1.12	4.32 ± 1.13	4.63 ± 1.26	<0.001
TG (mmol/L)	1.42 (0.87-2.30)	1.51 (1.03-2.36)	1.82 (1.18-2.81)	<0.001
HDL-C (mmol/L)	1.11 (0.92-1.35)	1.06 (0.88-1.25)	1.05 (0.86-1.26)	<0.001
LDL-C (mmol/L)	2.56 (2.00-3.16)	2.43 (1.99-3.09)	2.64 (2.09-3.27)	0.003
FBG (mmol/L)	5.09 (4.08-6.83)	6.87 (4.90-9.26)	5.41 (4.27-7.14)	<0.001
hs-CRP	10.13 (1.76-28.41)	7.45 (0.51-28.44)	5.41 (4.27-7.14)	0.009
Female (%)	1455 (68.57%)	127 (59.35%)	590 (61.78%)	<0.001
Hypertension (%)	627 (29.55%)	75 (35.05%)	447 (46.81%)	<0.001
Diabetes (%)	269 (12.68%)	66 (30.84%)	274 (28.69%)	<0.001
Hyperlipemia (%)	193 (9.10%)	28 (13.08%)	219 (22.93%)	<0.001
Pituitary tumor stroke (%)	86 (4.05%)	12 (5.61%)	37 (3.87%)	0.504
Aggressive (%)	317 (14.94%)	80 (37.38%)	235 (24.61%)	<0.001
Diabetes insipidus (%)	67 (3.16%)	16 (7.48%)	47 (4.92%)	0.002
Pituitary insufficiency (%)	634 (29.88%)	84 (39.25%)	955 (100.00%)	<0.001
Number of pituitary deficiencies				
0 (%)	1488 (70.12%)	130 (60.75%)	0 (0.00%)	
1 (%)	483 (22.76%)	60 (28.04%)	260 (27.23%)	<0.001
2 (%)	139 (6.55%)	22 (10.28%)	562 (58.85%)	
3 (%)	12 (0.57%)	2 (0.93%)	83 (8.69%)	
4 (%)	0 (0.00%)	0 (0.00%)	50 (5.24%)	
Gonad axis				
Normal (%)	1860 (87.65%)	183 (85.51%)	626 (65.55%)	
Hyperactivity (%)	10 (0.47%)	4 (1.87%)	2 (0.21%)	<0.001
Insufficiency (%)	252 (11.88%)	27 (12.62%)	327 (34.24%)	
Thyroid axis				
Normal (%)	1774 (83.60%)	167 (78.04%)	618 (64.71%)	
Hyperactivity (%)	39 (1.84%)	1 (0.47%)	19 (1.99%)	<0.001
Insufficiency (%)	309 (14.56%)	46 (21.50%)	318 (33.30%)	
Adrenal axis				
Normal (%)	1870 (88.12%)	171 (79.91%)	563 (58.95%)	
Hyperactivity (%)	16 (0.75%)	6 (2.80%)	57 (5.97%)	<0.001
Insufficiency (%)	236 (11.12%)	37 (17.29%)	335 (35.08%)	
Tumor size				
Microadenoma (%)	1611 (75.92%)	102 (47.66%)	526 (55.08%)	
Macroadenoma (%)	247 (11.64%)	37 (17.29%)	100 (10.47%)	<0.001
Giant adenoma (%)	264 (12.44%)	75 (35.05%)	329 (34.45%)	
Tumor function				
Non-function (%)	1461 (68.85%)	0 (0.00%)	689 (72.15%)	
Function (%)	661 (31.15%)	214 (100.00%)	266 (27.85%)	<0.001
Treatment				
Surgery (%)	746 (35.16%)	147 (68.69%)	331 (34.66%)	<0.001
Gamma knife (%)	128 (6.03%)	8 (3.74%)	36 (3.77%)	0.020
Degree of tumor resection				
Subtotal resection (%)	363 (17.11%)	50 (23.36%)	195 (20.42%)	
Total resection (%)	383 (18.05%)	97 (45.33%)	136 (14.24%)	<0.001
Not operated (%)	1376 (64.84%)	67 (31.31%)	624 (65.34%)	

Data are mean (standard deviation), n (%), or median (interquartile range).

IGF-1, insulin-like growth factor I; PRL, prolactin; BMI, body mass index; UA, uric acid; TC, total cholesterol; TG, triglyceride; HDL-C, high-density lipoprotein cholesterol; LDL-C, low-density lipoprotein cholesterol; FBG, fasting blood glucose; hs-CRP, hypersensitive C-reactive protein.

### 3.2 Relationship between IGF-1 and new-onset NAFLD

**Figure 2:**
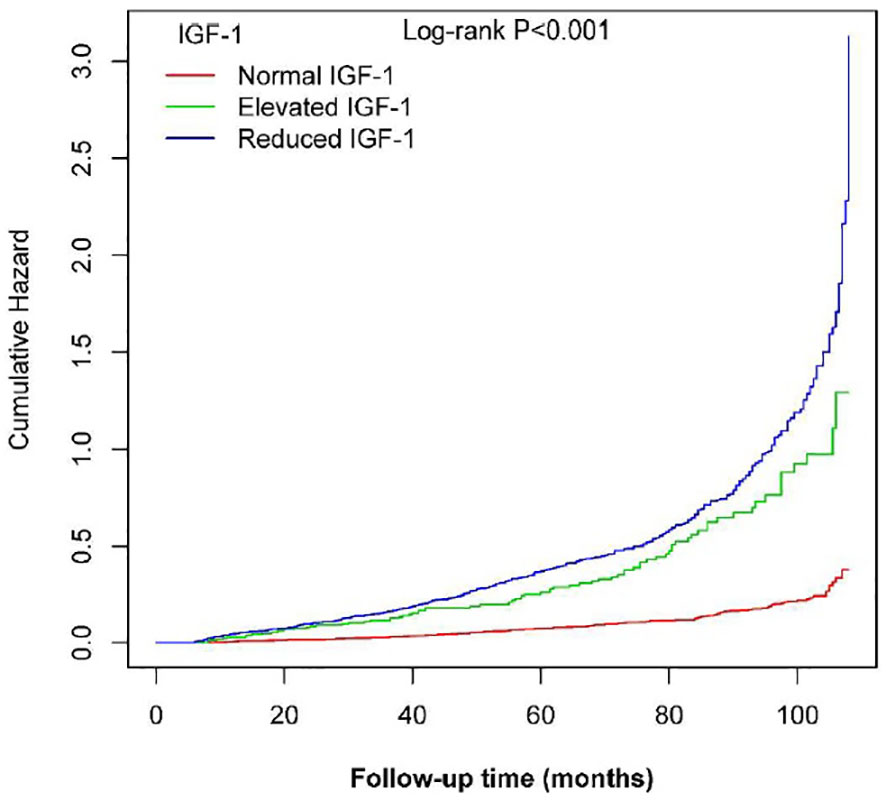
Kaplan-Meier curves showing the cumulative hazards of new-onset NAFLD based on categories in PitNET patients.

During a median follow-up of 65 months, 698 NAFLD events occurred. Kaplan-Meier curve graphs confirmed that patients in the group with reduced IGF-1 and elevated IGF-1 levels at baseline had a greater likelihood of suffering NAFLD compared to patients in the normal IGF-1 group (log-rank test, P < 0.001) (**Figure 2**). **Table 2** displays associations between IGF-1 levels and the risk of NAFLD. IGF-1 was evaluated as a ranked variable using age- and sex-adjusted reference ranges. In Cox regression analyses, we observed that both reduced and elevated IGF-1 levels were associated with an increased risk of NAFLD in non-adjusted and adjusted models. Compared with participants with normal IGF-1, the HRs of those with elevated and reduced IGF-1 were 2.33 (95% CI 1.75, 3.11) and 2.2 (95% CI 1.78, 2.7) in model IV, respectively.

**Table 2:** Effects of IGF-1 on new-onset NAFLD in individuals with PitNET.

Exposure	HR (95%CI) P-value				
	Non-adjusted	Model I	Model II	Model III	Model IV
IGF-1 categories					
Normal IGF-1	Reference	Reference	Reference	Reference	Reference
Elevated IGF-1	3.90 (3.00, 5.07) <0.001	3.55 (2.73, 4.62) <0.001	2.89 (2.21, 3.78) <0.001	2.12 (1.61, 2.81) <0.001	2.33 (1.75, 3.11) <0.001
Reduced IGF-1	5.29 (4.45, 6.28) <0.001	4.59 (3.84, 5.50) <0.001	2.62 (2.15, 3.20) <0.001	2.16 (1.76, 2.66) <0.001	2.20 (1.78, 2.70) <0.001

Non-adjusted adjust for none. Model I adjusts for age, sex, and BMI. Model II adjusts for the variables in Model I plus the gonad axis, thyroid axis, and adrenal axis. Model III adjusts for the variables in Model II plus tumor size, hypertension, diabetes, and hyperlipemia. Model IV adjusts for the variables in Model III plus aggressive, gamma knife, pituitary tumor stroke, PRL, UA, TC, TG, HDL-C, LDL-C, FBG, and hs-CRP.

IGF-1, insulin-like growth factor I; NAFLD, non-alcoholic fatty liver disease; PitNET, pituitary neuroendocrine tumors; HR, hazard ratio; BMI, body mass index; PRL, prolactin; UA, uric acid; TC, total cholesterol; TG, triglyceride; HDL-C, high-density lipoprotein cholesterol; LDL-C, low-density lipoprotein cholesterol; FBG, fasting blood glucose; hs-CRP, hypersensitive C-reactive protein.

### 3.3 Sensitivity analysis

We carried out the following sensitivity analyses. To minimize the reverse causation, we excluded instances that occurred within the first 2 years of follow-up, and the results remained consistent (**Supplementary Table 2**). Also, the relationship of IGF-1 with the risk of NAFLD development remained robust after removing patients with aggressive PitNET (**Supplementary Table 3**). Furthermore, reduced IGF-1 and elevated IGF-1 were still associated with a significantly higher risk of NAFLD after excluding patients with giant PitNET (**Supplementary Table 4**). Additionally, in patients with PitNET who underwent surgery, the association of IGF-1 with the risk of new-onset NAFLD was also observed (**Supplementary Table 5**). Likewise, the results remained similar after considering death as a competing risk (**Supplementary Table 6**). These sensitivity analyses did not materially alter the risk estimates.

### 3.4 Dose-response and threshold effect analysis

**Figure 3:**
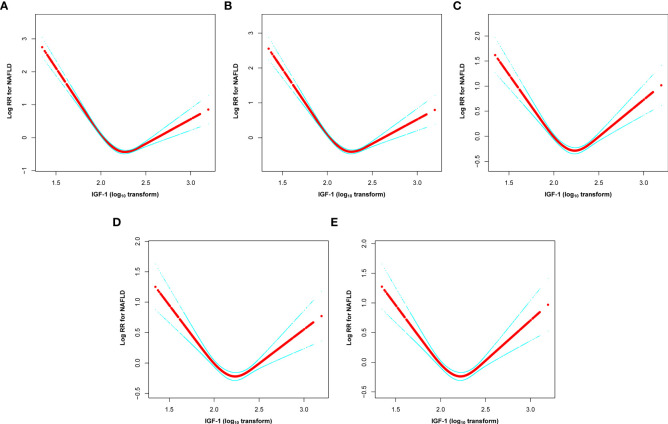
U-shaped relationship between lgIGF-1 and the risk of new-onset NAFLD in PitNET patients. **(A)** non-adjusted, **(B)** model I, **(C)** model II, **(D)** model III, and **(E)** model IV. Non-adjusted adjust for none. Model I adjusts for age, sex, and BMI. Model II adjusts for the variables in Model I plus the gonad axis, thyroid axis, and adrenal axis. Model III adjusts for the variables in Model II plus tumor size, hypertension, diabetes, and hyperlipemia. Model IV adjusts for the variables in Model III plus aggressive, gamma knife, pituitary tumor stroke, PRL, UA, TC, TG, HDL-C, LDL-C, FBG, and hs-CRP. IGF-1, insulin-like growth factor I; NAFLD, non-alcoholic fatty liver disease; PitNET, pituitary neuroendocrine tumors; HR, hazard ratio; BMI, body mass index; PRL, prolactin; UA, uric acid; TC, total cholesterol; TG, triglyceride; HDL-C, high-density lipoprotein cholesterol; LDL-C, low-density lipoprotein cholesterol; FBG, fasting blood glucose; hs-CRP, hypersensitive C-reactive protein.

The dose-response and threshold effects between IGF-1 levels and the risk of NAFLD in PitNET patients are shown in **Table 3** and **Figure 3**. The association between IGF-1 levels and NAFLD rates followed a U-shaped curve, with both reduced and elevated IGF-1 levels significantly correlating with higher rates of NAFLD. To elucidate the nonlinear relationship, a recursive algorithm and two-piecewise linear analysis were utilized to identify the inflection point of lgIGF-1, which was determined to be 2.35 ng/ml (log likelihood ratio test, P< 0.001). This U-shaped relationship between IGF-1 levels and the risk of NAFLD persisted even after adjustments in multiple models. For lgIGF-1 levels below 2.35 ng/ml, the HR for NAFLD was 0.24 (95% CI 0.15-0.37) in model IV. Conversely, for lgIGF-1 levels above 2.35 ng/ml, the HR for NAFLD was 7.8 (95% CI 4.01-15.17) in model IV.

**Table 3:** Threshold effect analyses of lg_10_(IGF-1) on the risk of new-onset NAFLD using two-piecewise regression models.

Risk of developing NAFLD	HR (95%CI) P-value				
	Non-adjusted	Model I	Model II	Model III	Model IV
Fitting by the standard linear model	0.19 (0.14, 0.25) <0.001	0.25 (0.18, 0.34) <0.001	0.62 (0.46, 0.84) 0.002	0.73 (0.55, 0.98) 0.036	0.76 (0.56, 1.03) 0.078
Fitting by the two-piecewise linear model					
Inflection point	2.35	2.35	2.35	2.35	2.35
Lg_10_(IGF-1) < 2.35ng/ml	0.04 (0.03, 0.06) <0.001	0.05 (0.04, 0.08) <0.001	0.16 (0.11, 0.24) <0.001	0.16 (0.11, 0.24) <0.001	0.24 (0.15, 0.37) <0.001
Lg_10_(IGF-1)> 2.35ng/ml	28.57 (15.93, 51.25) <0.001	17.2 (9.62, 30.77) <0.001	10.77 (6.02, 19.27) <0.001	10.77 (6.02, 19.27) <0.001	7.8 (4.01, 15.17) <0.001
P for log-likelihood ratio test	<0.001	<0.001	<0.001	<0.001	<0.001

Non-adjusted adjust for none. Model I adjusts for age, sex, and BMI. Model II adjusts for the variables in Model I plus the gonad axis, thyroid axis, and adrenal axis. Model III adjusts for the variables in Model II plus tumor size, hypertension, diabetes, and hyperlipemia. Model IV adjusts for the variables in Model III plus aggressive, gamma knife, pituitary tumor stroke, PRL, UA, TC, TG, HDL-C, LDL-C, FBG, and hs-CRP.

IGF-1, insulin-like growth factor I; NAFLD, non-alcoholic fatty liver disease; PitNET, pituitary neuroendocrine tumors; HR, hazard ratio; BMI, body mass index; PRL, prolactin; UA, uric acid; TC, total cholesterol; TG, triglyceride; HDL-C, high-density lipoprotein cholesterol; LDL-C, low-density lipoprotein cholesterol; FBG, fasting blood glucose; hs-CRP, hypersensitive C-reactive protein.

### 3.5 Stratification analysis and tests for interaction

**Figure 4:**
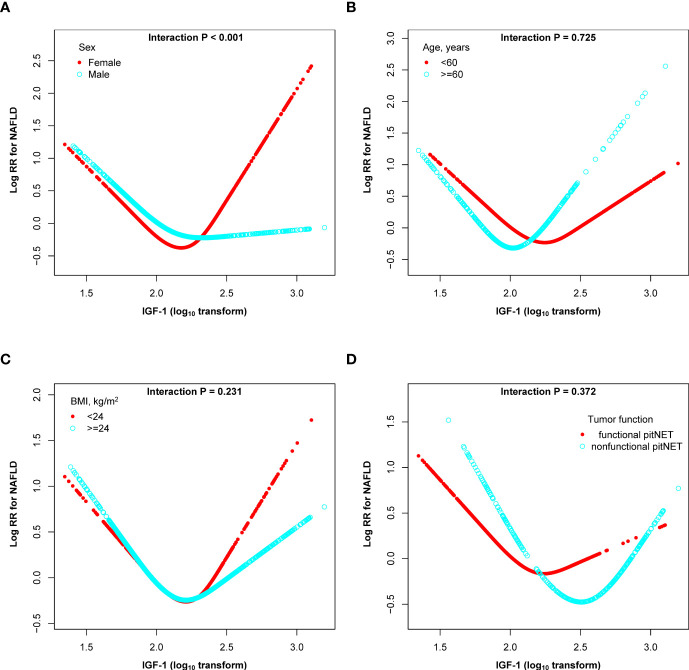
Subgroup analyses of the association between the lgIGF-1 and the risk of NAFLD in PitNET patients. **(A)** subgroup analyses stratified by sex; **(B)** subgroup analyses stratified by sex; **(C)** subgroup analyses stratified by BMI; **(D)** subgroup analyses stratified by tumor function. Adjusted, if not stratified, for age, sex, BMI, gonad axis, thyroid axis, adrenal axis, tumor size, hypertension, diabetes, hyperlipemia, aggressive, gamma knife, pituitary tumor stroke, PRL, UA, TC, TG, HDL-C, LDL-C, FBG, and hs-CRP.

We conducted stratified analyses to assess the relationship between IGF-1 and the incidence of NAFLD in various subgroups (**Figure 4**). Subgroup tests revealed that sex had an interaction effect on the relationship between IGF-1 and NAFLD (P interaction < 0.001). In contrast, the association between IGF-1 and incidence of NAFLD was not significantly modified by age, BMI and tumor function (P interactions > 0.05).

## 4 Discussion

In this study, we found a U-shaped relationship between IGF-1 and the risk of new-onset NAFLD in patients with PitNET. After adjusting for potential confounders, both reduced and elevated IGF-1 levels were strongly associated with an increased risk of NAFLD compared with patients with normal IGF-1 levels. These findings remained robust in sensitivity analyses and were consistent across all subgroups.

Previous studies have confirmed that IGF-1 levels are reduced in obese populations and that the relative deficiency of GH and IGF-1 in obese patients promotes the development of NAFLD (27, 31). Randomized controlled trials have demonstrated that recombinant human growth hormone (rhGH) replacement therapy significantly improves hepatic steatosis in obese adolescents and adults (22). In a study by Nishizawa et al, the incidence of NAFLD was higher in GHD patients than in controls, and hepatic steatosis and fibrosis were reduced 6 months after rhGH therapy (32). A cross-sectional study suggested that IGF-1 deficiency was positively associated with NAFLD (defined using a validated hepatic steatosis index) in patients with PitNET (33). However, another study did not find an association between IGF-1 deficiency and NAFLD (estimated by calculating the fatty liver index) in patients with non-functioning PitNET (34). It is noteworthy that the two studies defined NAFLD differently. In our current study, NAFLD was defined using abdominal ultrasonography. Besides, the cross-sectional designs of these studies hindered the evaluation of causality between IGF-1 deficiency and NAFLD.

IGF-1 deficiency is associated with dyslipidemia, visceral obesity, weight gain, and insulin resistance (23), all of which are involved in the progression of NAFLD (35–37). In the presence of IGF-1 deficiency, multiple mechanisms contribute to the development of NAFLD. Firstly, previous studies have shown an association between IGF-1 deficiency, liver dysfunction and hyperlipidemia (38). Furthermore, recent studies have confirmed that rhGH treatment improves lipid metabolism in obese children (39, 40). In our study, we also found higher levels of blood lipids in the group with reduced IGF-1 levels. Secondly, excess lipid accumulation in the liver plays a crucial role in hepatic steatosis (41, 42). IGF-1, as the effector hormone of GH, mediates its protective role in the pathogenesis of NAFLD by regulating cholesterol transport and regulating the adipogenic pathway (43). Furthermore, IGF-1 is responsible for regulating lipolysis and anti-inflammatory responses (44). Deficiency of GH or IGF-1 leads to the absence of feedback loops in the hypothalamic-pituitary-adipose axis (45). Thirdly, deficiency of the GH/IGF-1 system produces leptin resistance, which leads to bulimia, obesity and subsequent insulin resistance, all of which contribute to early and aggressive hepatic steatosis (46). Lastly, the regulation of metabolic, immune and hepatic stellate cell function by IGF-1 is crucial in preventing the development of NAFLD (43). Patients with pituitary tumors often experience hypopituitarism due to the occupational effects of the tumor (16), with GH/IGF system involvement being the most common (17). Overall, these prior studies suggest that maintaining optimal IGF-1 levels through GH replacement therapy may be an effective treatment option for GHD patients with liver impairment and associated steatosis and fibrosis.

In our study, elevated IGF-1 was also an established risk factor for NAFLD. Functional GH-secreting pituitary adenomas, also known as acromegaly, are characterized by increased levels of growth hormone and its target hormone, IGF-1 (47). According to guidelines, IGF-1 is recommended as a screening and diagnostic tool for acromegaly (48). As indicated by multiple studies, there is a correlation between acromegaly and metabolic disorders, such as hypertension, diabetes, and dyslipidemia (49). While glucose and lipid abnormalities, as well as metabolic disorders, are frequently observed in patients with acromegaly, most cases can be effectively managed by normalizing IGF-1 levels (50–52). However, the prevalence of NAFLD in patients suffering from acromegaly and the correlation between elevated IGF-1 levels and NAFLD have not been investigated. In our current study, we observed that individuals with elevated IGF-1 levels had a 2fold higher risk of NAFLD compared to those with normal IGF-1 levels, after adjusting for covariates. This suggests that controlling IGF-1 levels within the standard range can decrease the incidence of NAFLD in individuals diagnosed with acromegaly. Achieving biochemical remission is one of the therapeutic goals for patients with acromegaly, and guidelines recommend maintaining serum IGF-1 within the sex- and age-specific normal ranges (53). Studies have shown that biochemical control of acromegaly can normalize mortality rates (49).

Our findings suggest that reduced and elevated IGF-1 levels are associated with an increased risk of new-onset NAFLD. This further emphasizes the importance of maintaining IGF-1 in an optimal range to prevent NAFLD in patients with PitNET. Furthermore, the dose-response and threshold effect analyses presented in our study propose a potential cut-off point that could guide the establishment of targeted IGF-1 level management in patients with PitNET. However, this proposed threshold warrants validation through additional prospective research. In the future, exploring the association between IGF-1 levels and the risk of NAFLD in a broader demographic could validate the necessity for IGF-1 level screening in populations vulnerable to NAFLD, potentially leading to more precise identification of individuals at increased risk for the condition. The potential use of GH, IGF-1 modulators, or other agents that directly target the IGF-1 axis as therapeutic options for NAFLD remains an intriguing avenue for future exploration. Additionally, examining the relationship between IGF-1 and NAFLD through genetic, molecular, and environmental lenses could yield novel preventive and management strategies for NAFLD. The public health significance of these findings is profound, given the rising incidence of NAFLD in the global population.

## 5 Strengths and limitations

There are several strengths in our study, including the large sample size, long follow-up time, low loss to follow-up rate, and strict statistical analysis. Despite these strengths, some limitations of this study are important to note. Firstly, the patient population was collected retrospectively from a single institution. Additional studies of a larger patient cohort are clearly needed to validate and refine these findings. However, it is worth noting that PitNET is not a common disease, making it challenging to conduct prospective studies on a large scale. Secondly, we cannot rule out the possibility of some residual confounding despite a comprehensive adjustment of baseline characteristics. Thirdly, causality cannot be confirmed in this study due to its retrospective design. However, to mitigate the potential impact of reverse causality, patients with preexisting NAFLD were excluded from the study. Fourth, the levels of IGF-1 reported in this study are based on a single measurement taken at baseline, which may not adequately reflect the dynamic changes that occur during long-term follow-up. Fifth, based on the instability of GH and the large number of missing GH data, our study did not identify the association between GH and NAFLD.

## 6 Conclusion

In summary, in our cohort study, there was a U-shaped trend between IGF-1 and new-onset NAFLD in patients with PitNET. Both elevated and reduced levels of IGF-1 were associated with an increased risk of NAFLD. It is suggested that individualized, comprehensive treatment of PitNET might reduce the occurrence of comorbidities. Thus, further studies are necessary to confirm our findings.

## Data availability statement

The data analyzed in this study is subject to the following licenses/restrictions: Some or all of the datasets generated and/or analyzed during the current study are not publicly available but can be obtained from the corresponding author upon reasonable request. Requests to access these datasets should be directed to guozeyang@126.com.

## Ethics statement

The studies involving humans were approved by Ethical Committee of the People’s Hospital of Xinjiang Uygur Autonomous Region. The studies were conducted in accordance with the local legislation and institutional requirements. Written informed consent for participation was not required from the participants or the participants’ legal guardians/next of kin because as a retrospective cohort study, ethical approval is not necessary.

## Author contributions

YG: Methodology, Writing – review & editing. YH: Methodology, Writing – original draft. CY: Methodology, Writing – review & editing. MA: Software, Writing – review & editing. JM: Data curation, Writing – review & editing. ZJ: Project administration, Validation, Writing – review & editing. AT: Formal analysis, Methodology, Writing – review & editing. HA: Software, Writing – review & editing.
