# Supplementary material for: U-Shaped relationship of insulin-like growth factor I and incidence of nonalcoholic fatty liver in patients with pituitary neuroendocrine tumors: a cohort study
Source: Front Endocrinol (Lausanne). 2024 Feb 2;15:1290007. doi: 10.3389/fendo.2024.1290007 (PMC10869555; doi:10.3389/fendo.2024.1290007)
Supplement: Supplementary file 1 [file DataSheet_1.docx]

Supplementary Material

# Supplemental material and methods

## Baseline examination

Anthropometric measurements were also taken by trained staff at baseline. Data for height and weight were acquired following a protocol standardized to an accuracy of 0.1 kg and 0.1 cm, respectively. During the measurements, participants were asked to wear light clothing, no hats, and no shoes. The BMI is calculated as weight in kilograms divided by height in meters squared.

Alcohol consumption was evaluated with questions regarding the types of alcoholic beverages, the frequency of alcohol consumption per week, and the usual amount consumed per occasion. Subjects who reported alcohol consumption >140 g/week for men and >70 g/week for women were deemed to have excessive alcohol consumption [1].

Information on disease history was obtained using the International Classification of Diseases, 10th Revision (ICD-10) codes. Diabetes was indicated by codes E10-E14, hypertension was indicated by code I10, hyperlipemia was indicated by code E78, NAFLD was indicated by code K76, and pituitary neuroendocrine tumors (PitNET) were indicated by code D35.2.

Aggressive pituitary neuroendocrine tumors (PitNET) can be diagnosed by the presence of at least one of the following: (1) Knosp grades 3 or 4; (2) invasion of the sphenoid sinus documented at surgery or in pathological analysis; (3) growth >20% and at least 2 mm within 6 months or growth >20% despite adequate surgery, medical treatment, and radiotherapy [2].

PitNET can be described as microadenomas, macroadenomas, or giant tumors based on size. Microadenoma refers to a tumor smaller than 10 mm, while macroadenoma describes a tumor larger than 10mm. Giant PitNET are bigger than 40 mm [3].

## Definition of insufficiency

Hypopituitarism was diagnosed based on morning hormone levels and stimulating tests. Central hypothyroidism was defined as low serum-free thyroxine levels associated with an inappropriately low or normal level of thyroid-stimulating hormone (TSH) [4]. Hypothalamic-pituitary-adrenal axis hypoplasia was defined as a morning basal cortisol (8 a.m.) less than 138 nmol/L associated with an inappropriately low or normal adrenocorticotropic hormone (ACTH) level [5]. In male patients, hypogonadism secondary was defined as delayed or absent pubertal development with low serum testosterone (T < 3.47 nmol/L) associated with inappropriately low or normal luteinizing hormone (LH) and follicle-stimulating hormone (FSH) levels [6]. Female patients were considered to have secondary hypogonadism if they had irregular menstruation cycles or amenorrhea and the follicle-stimulating hormone level was less than 40 IU/L; otherwise, they were considered to be postmenopausal [7].The diagnosis of central diabetes insipidus was confirmed when patients presented with polyuria (urinary volume >3 L/d) and a urine osmolarity <600 mOsmol/L in the morning urine sample when the serum osmolarity was >295 mOsmol/L [4].

## Definition of hyperactivity

Serum thyrotropin and free thyroxine levels are measured using immunoassays. If serum free thyroxine is elevated and thyrotropin is high or inappropriately normal, the pituitary-thyroid axis is considered hyperactive [8]. Hyperactivity of the gonad axis is defined as elevated or non-suppressed FSH and suppressed or inappropriately “normal” LH in the setting of marginally, moderately, or extremely elevated estradiol; continuously and remarkably elevated LH [9]. Hyperactivity of the adrenal axis is defined as high or inadequate normal levels of ACTH in the presence of high late-night salivary cortisol levels, high urinary free cortisol levels or unsuppressed levels of cortisol at dexamethasone suppression tests [10].

## Assessment of NAFLD

NAFLD was diagnosed by abdominal ultrasonography. Each participant's abdominal ultrasonography was performed by experienced radiologists using a B-mode topographical ultrasound system with a 3.5 MHz probe (Acuson X300, Siemens, Munich, Germany). After excluding other causes of liver diseases and excessive alcohol intake, NAFLD can be diagnosed by the presence of at least two of the following abnormalities on abdominal ultrasonography: (1) increased echogenicity compared to the spleen and kidneys, indicating diffuse hyperechogenicity; (2) diminished visualization of specific liver structures; (3) slight to moderate hepatomegaly with bluntly rounded margins; (4) diminished hepatic perfusion with maintained distribution of blood flow; and (5) indistinct visualization of the right hepatic lobe and diaphragmatic margin [11].

## Drug therapy

Patients with adrenal insufficiency were treated with conventional hydrocortisone, usually divided into 2-3 doses per day. Secondary hypothyroidism was treated with levothyroxine. Replacement therapy for gonadotropin deficiency is administered to treat hypogonadism in premenopausal women and in men who are younger than 65 years old. In the case of older men and postmenopausal women, the decision to initiate replacement therapy is tailored to the individualized assessment. IGF-1 deficiency was not treated with replacement in this study. Drug therapy for functional PitNET administered as follows: 73 patients were treated with octreotide, of which 69 had acromegaly and 4 had Cushing's disease. Bromocriptine was prescribed to 635 patients with prolactinomas. No patients were treated with pegvisomant, as it was not available.

## Statistical analysis

IGF-1 at baseline was separated into three groups according to age- and sex-adjusted reference ranges: normal IGF-1, elevated IGF-1, and reduced IGF-1. Clinical and demographic characteristics were reported using proportions, the mean and standard deviation (SD), or the median and interquartile range (IQR), as appropriate. To compare the characteristics among different IGF-1 groups, the chi-square test was performed for categorical variables, and the one-way analysis of variance, or Kruskal-Wallis test, was performed for continuous variables with normal and skewed distributions.

The Kaplan-Meier method was performed to evaluate the incidence rate of NAFLD, and the differences among groups were evaluated using the log-rank test. To assess for collinearity, we measured the variance inflation factor (VIF) in all models using a predetermined threshold of 5 as suggestive of multicollinearity (Table S1). Variables with VIFs above 5 were removed. A Cox proportional hazard regression model was applied to calculate the hazard ratios (HRs) and 95% confidence intervals (CIs) for NAFLD. IGF-1 was assessed as a categorical and continuous variable. Given a non-normal distribution, IGF-1 was lg-transformed for the continuous model. We constructed three distinct models: the non-adjusted model (adjust for none of the variables), model I (adjust for age, sex, and BMI), model II (adjust for the variables in Model I plus the gonad axis, thyroid axis, and adrenal axis), model III (adjust for the variables in Model II plus tumor size, hypertension, diabetes, and hyperlipemia), and model IV (adjust for the variables in Model III plus aggressive, gamma knife, pituitary tumor stroke, PRL, UA, TC, TG, HDL-C, LDL-C, FBG, and hs-CRP).

Non-linear relationships were checked by fitting a generalized additive model (GAM) between the independent and dependent variables, featuring a nonlinear smoother, in the R package mgcv. If a non-linear association was observed, a two-piecewise linear regression model was performed to calculate the threshold effect of lgIGF-1 on the incidence of NAFLD based on the smoothing plot. The recursive method automatically calculated the inflection point by using the maximum model likelihood when the inflection point appeared in the curve.

Furthermore, to investigate whether the association between IGF-1 and the incidence of NAFLD (the main outcome) differed by population subgroup, the analyses were repeated and stratified by sex (male and female), age (≥60 and < 60 years), BMI(≥24 and < 24 kg/m^2^), and tumor function (functional PitNET and nonfunctional PitNET).

We also conducted a series of sensitivity analyses to ensure the robustness of our findings. First, in order to minimize the reverse causality, we excluded the cases that occurred within the first 2 years of follow-up. Second, we restricted analyses in patients with non-aggressive PitNET. Third, sensitivity analyses were conducted by sequentially adjusting for PitNET tumor size. Fourth, we analyzed whether the association would change if only PitNET individuals who underwent surgery were selected. Fifth, as an additional sensitivity analysis, models were assessed using a competing risk regression, considering all-cause mortality as a competing risk, using the method of Fine and Gray.

A 2-tailed P < 0.05 was considered to be statistically significant in all analyses. R software, version 4.1.1 (R Project for Statistical Computing), was used for all data analyses.

# Supplementary figures and tables

## Supplementary tables

**Table S1.** The variance inflation factor (VIF) of all preliminary variables in the ordinary least squares (OLS) model.

| Variable name | Step 1 | Step 2 |
| --- | --- | --- |
| IGF-1 | 2.3 | 1.7 |
| Age | 1.5 | 1.4 |
| Sex | 1.4 | 1.4 |
| Tumor size | 1.4 | 1.4 |
| Degree of tumor resection | 7.6 | NA |
| Aggressive | 1.5 | 1.5 |
| Surgery | 8.4 | NA |
| Gamma knife | 1.1 | 1.1 |
| Pituitary tumor stroke | 1.1 | 1.1 |
| Hypertension | 1.3 | 1.3 |
| Diabetes | 1.6 | 1.6 |
| Hyperlipemia | 1.2 | 1.2 |
| Pituitary insufficiency | 3.6 | 2.6 |
| Number of pituitary deficiencies | 12.6 | NA |
| Diabetes insipidus | 1.1 | 1.1 |
| PRL | 1.1 | 1.1 |
| Gonad axis | 3.3 | 1.6 |
| Thyroid axis | 2.5 | 1.5 |
| Adrenal axis | 2.9 | 1.6 |
| Tumor function | 6.3 | NA |
| BMI | 1.2 | 1.2 |
| UA | 1.3 | 1.3 |
| TC | 6.5 | NA |
| TG | 2.3 | 2.3 |
| HDL | 1.6 | 1.5 |
| LDL | 4.8 | 4.8 |
| FBG | 1.7 | 1.7 |
| hs-CRP | 1.3 | 1.3 |

VIF = 1/(1-R2). VIF step-by-step screening method: Calculate the VIF of each variable. If the maximum VIF value >10, remove the variable with the maximum VIF value.

**Abbreviations:** IGF-1, insulin-like growth factor I; PRL, prolactin; BMI, body mass index; UA, uric acid; TC, total cholesterol; TG, triglyceride; HDL-C, high-density lipoprotein cholesterol; LDL-C, low-density lipoprotein cholesterol; FBG, fasting blood glucose; hs-CRP, hypersensitive C-reactive protein; NAFLD.

**Table S2.** Cox proportional hazards model analysis of the incidence of NAFLD in different groups (sensitivity analysis: excluding the cases that occurred within the first 2 years of follow-up)

| Exposure | HR (95%CI) P-value | | | | |
| --- | --- | --- | --- | --- | --- |
|  | Non-adjusted | Model I | Model II | Model III | Model IV |
| IGF-1 categories |  |  |  |  |  |
| Normal IGF-1 | Reference | Reference | Reference | Reference | Reference |
| Elevated IGF-1 | 3.71 (2.77, 4.97) <0.001 | 3.37 (2.51, 4.52) <0.001 | 2.77 (2.05, 3.73) <0.001 | 2.08 (1.52, 2.84) <0.001 | 2.31 (1.68, 3.19) <0.001 |
| Reduced IGF-1 | 5.15 (4.26, 6.23) <0.001 | 4.37 (3.58, 5.34) <0.001 | 2.68 (2.15, 3.34) <0.001 | 2.24 (1.78, 2.82) <0.001 | 2.29 (1.82, 2.88) <0.001 |

**Notes**: Non-adjusted adjust for none. Model I adjusts for age, sex, and BMI. Model II adjusts for the variables in Model I plus the gonad axis, thyroid axis, and adrenal axis. Model III adjusts for the variables in Model II plus tumor size, hypertension, diabetes, and hyperlipemia. Model IV adjusts for the variables in Model III plus aggressive, gamma knife, pituitary tumor stroke, PRL, UA, TC, TG, HDL-C, LDL-C, FBG, and hs-CRP.

**Abbreviations**: IGF-1, insulin-like growth factor I; NAFLD, non-alcoholic fatty liver disease; HR, Hazard ratio; BMI, body mass index; PRL, prolactin; UA, uric acid; TC, total cholesterol; TG, triglyceride; HDL-C, high-density lipoprotein cholesterol; LDL-C, low-density lipoprotein cholesterol; FBG, fasting blood glucose; hs-CRP, hypersensitive C-reactive protein.

**Table S3.** Cox proportional hazards model analysis of the incidence of NAFLD in different groups (sensitivity analysis: patients with aggressive PitNET were excluded).

**Notes:** Non-adjusted adjust for none. Model I adjusts for age, sex, and BMI. Model II adjusts for the variables in Model I plus the gonad axis, thyroid axis, and adrenal axis. Model III adjusts for the variables in Model II plus tumor size, hypertension, diabetes, and hyperlipemia. Model IV adjusts for the variables in Model III plus gamma knife, pituitary tumor stroke, PRL, UA, TC, TG, HDL-C, LDL-C, FBG, and hs-CRP.

**Abbreviations:** IGF-1, insulin-like growth factor I; NAFLD, non-alcoholic fatty liver disease; HR, Hazard ratio; BMI, body mass index; PRL, prolactin; UA, uric acid; TC, total cholesterol; TG, triglyceride; HDL-C, high-density lipoprotein cholesterol; LDL-C, low-density lipoprotein cholesterol; FBG, fasting blood glucose; hs-CRP, hypersensitive C-reactive protein.

**Table S4.** Cox proportional hazards model analysis of the incidence of NAFLD in different groups (sensitivity analysis: patients with giant PitNET were excluded).

| Exposure | HR (95%CI) P-value | | | | |
| --- | --- | --- | --- | --- | --- |
|  | Non-adjusted | Model I | Model II | Model III | Model IV |
| IGF-1 categories |  |  |  |  |  |
| Normal IGF-1 | Reference | Reference | Reference | Reference | Reference |
| Elevated IGF-1 | 4.23 (3.03, 5.90) <0.001 | 4.01 (2.87, 5.61) <0.001 | 3.34 (2.38, 4.68) <0.001 | 2.08 (1.52, 2.84) <0.001 | 2.23 (1.61, 3.07) <0.001 |
| Reduced IGF-1 | 6.72 (5.46, 8.27) <0.001 | 5.83 (4.69, 7.24) <0.001 | 3.47 (2.74, 4.41) <0.001 | 2.24 (1.78, 2.82) <0.001 | 2.23 (1.77, 2.81) <0.001 |

| Exposure | HR (95%CI) P-value | | | | |
| --- | --- | --- | --- | --- | --- |
|  | Non-adjusted | Model I | Model II | Model III | Model IV |
| IGF-1 categories |  |  |  |  |  |
| Normal IGF-1 | Reference | Reference | Reference | Reference | Reference |
| Elevated IGF-1 | 4.73 (3.44, 6.51) <0.001 | 4.31 (3.13, 5.95) <0.001 | 2.97 (2.14, 4.12) <0.001 | 2.91 (2.09, 4.05) <0.002 | 3.03 (2.15, 4.26) <0.001 |
| Reduced IGF-1 | 4.22 (3.42, 5.21) <0.001 | 3.61 (2.89, 4.51) <0.001 | 1.82 (1.42, 2.33) <0.001 | 1.57 (1.21, 2.02) 0.001 | 1.54 (1.19, 1.99) 0.001 |

**Notes:** Non-adjusted adjust for none. Model I adjusts for age, sex, and BMI. Model II adjusts for the variables in Model I plus the gonad axis, thyroid axis, and adrenal axis. Model III adjusts for the variables in Model II plus hypertension, diabetes, and hyperlipemia. Model IV adjusts for the variables in Model III plus aggressive, gamma knife, pituitary tumor stroke, PRL, UA, TC, TG, HDL-C, LDL-C, FBG, and hs-CRP.

**Abbreviations:** IGF-1, insulin-like growth factor I; NAFLD, non-alcoholic fatty liver disease; HR, Hazard ratio; BMI, body mass index; PRL, prolactin; UA, uric acid; TC, total cholesterol; TG, triglyceride; HDL-C, high-density lipoprotein cholesterol; LDL-C, low-density lipoprotein cholesterol; FBG, fasting blood glucose; hs-CRP, hypersensitive C-reactive protein.

**Table S5.** Cox proportional hazards model analysis of the incidence of NAFLD in different groups (sensitivity analysis: never surgically treated patients were excluded).

| Exposure | HR (95%CI) P-value | | | | |
| --- | --- | --- | --- | --- | --- |
|  | Non-adjusted | Model I | Model II | Model III | Model IV |
| IGF-1 categories |  |  |  |  |  |
| Normal IGF-1 | Reference | Reference | Reference | Reference | Reference |
| Elevated IGF-1 | 2.58 (1.84, 3.61) <0.001 | 2.54 (1.80, 3.57) <0.001 | 2.21 (1.55, 3.15) <0.001 | 1.67 (1.15, 2.44) 0.007 | 1.80 (1.20, 2.70) 0.005 |
| Reduced IGF-1 | 3.20 (2.45, 4.17) <0.001 | 2.99 (2.27, 3.95) <0.001 | 1.55 (1.14, 2.12)  0.006 | 1.27 (0.91, 1.76) 0.154 | 1.24 (0.89, 1.73) 0.209 |

**Notes**: Non-adjusted adjust for none. Model I adjusts for age, sex, and BMI. Model II adjusts for the variables in Model I plus the gonad axis, thyroid axis, and adrenal axis. Model III adjusts for the variables in Model II plus tumor size, hypertension, diabetes, and hyperlipemia. Model IV adjusts for the variables in Model III plus aggressive, gamma knife, pituitary tumor stroke, PRL, UA, TC, TG, HDL-C, LDL-C, FBG, and hs-CRP.

**Abbreviations**: IGF-1, insulin-like growth factor I; NAFLD, non-alcoholic fatty liver disease; HR, Hazard ratio; BMI, body mass index; PRL, prolactin; UA, uric acid; TC, total cholesterol; TG, triglyceride; HDL-C, high-density lipoprotein cholesterol; LDL-C, low-density lipoprotein cholesterol; FBG, fasting blood glucose; hs-CRP, hypersensitive C-reactive protein.

**Table S6.** Cox proportional hazards model analysis of the incidence of NAFLD in different groups (competing risk model for death)

| Exposure | SHR (95%CI) P-value | | | | |
| --- | --- | --- | --- | --- | --- |
|  | Non-adjusted | Adjust I | Adjust II | Adjust III | Adjust IV |
| IGF-1 categories |  |  |  |  |  |
| Normal IGF-1 | Reference | Reference | Reference | Reference | Reference |
| Elevated IGF-1 | 4.50 (3.44, 5.89) <0.001 | 4.16 (3.18, 5.45) <0.001 | 3.84 (2.93, 5.05) <0.001 | 2.79 (2.10, 3.71) <0.001 | 3.07 (2.28, 4.12) <0.001 |
| Reduced IGF-1 | 5.19 (4.35, 6.20) <0.001 | 4.53 (3.77, 5.46) <0.001 | 2.76 (2.25, 3.39) <0.001 | 2.22 (1.79, 2.75) <0.001 | 2.26 (1.82, 2.80) <0.001 |

**Notes**: Non-adjusted adjust for none. Model I adjusts for age, sex, and BMI. Model II adjusts for the variables in Model I plus the gonad axis, thyroid axis, and adrenal axis. Model III adjusts for the variables in Model II plus tumor size, hypertension, diabetes, and hyperlipemia. Model IV adjusts for the variables in Model III plus aggressive, gamma knife, pituitary tumor stroke, PRL, UA, TC, TG, HDL-C, LDL-C, FBG, and hs-CRP.

**Abbreviations**: IGF-1, insulin-like growth factor I; NAFLD, non-alcoholic fatty liver disease; SHR, Subdistribution hazard ratio; BMI, body mass index; PRL, prolactin; UA, uric acid; TC, total cholesterol; TG, triglyceride; HDL-C, high-density lipoprotein cholesterol; LDL-C, low-density lipoprotein cholesterol; FBG, fasting blood glucose; hs-CRP, hypersensitive C-reactive protein.

**References**

1. Farrell GC, Chitturi S, Lau GK, Sollano JD, Asia-Pacific Working Party on N. 2007 Guidelines for the assessment and management of non-alcoholic fatty liver disease in the Asia-Pacific region: executive summary. J Gastroenterol Hepatol.22(6):775-7. doi:10.1111/j.1440-1746.2007.05002.x.

2. Kasuki L, Raverot G. 2020 Definition and diagnosis of aggressive pituitary tumors. Rev Endocr Metab Disord.21(2):203-8. doi:10.1007/s11154-019-09531-x.

3. Russ S, Anastasopoulou C, Shafiq I. Pituitary Adenoma. StatPearls. Treasure Island (FL) ineligible companies. Disclosure: Catherine Anastasopoulou declares no relevant financial relationships with ineligible companies. Disclosure: Ismat Shafiq declares no relevant financial relationships with ineligible companies.2023.

4. Huang Q, Xu H, Wang X, Mao J, Yu B, Zhu Y et al. 2022 Relationship between growth hormone deficiency and nonalcoholic fatty liver disease in patients with pituitary stalk interruption syndrome. Clin Endocrinol (Oxf).97(5):612-21. doi:10.1111/cen.14732.

5. Kazlauskaite R, Evans AT, Villabona CV, Abdu TA, Ambrosi B, Atkinson AB et al. 2008 Corticotropin tests for hypothalamic-pituitary- adrenal insufficiency: a metaanalysis. J Clin Endocrinol Metab.93(11):4245-53. doi:10.1210/jc.2008-0710.

6. Zheng J, Mao J, Xu H, Wang X, Huang B, Liu Z et al. 2017 Pulsatile GnRH Therapy May Restore Hypothalamus-Pituitary-Testis Axis Function in Patients With Congenital Combined Pituitary Hormone Deficiency: A Prospective, Self-Controlled Trial. J Clin Endocrinol Metab.102(7):2291-300. doi:10.1210/jc.2016-3990.

7. Fleseriu M, Hashim IA, Karavitaki N, Melmed S, Murad MH, Salvatori R et al. 2016 Hormonal Replacement in Hypopituitarism in Adults: An Endocrine Society Clinical Practice Guideline. J Clin Endocrinol Metab.101(11):3888-921. doi:10.1210/jc.2016-2118.

8. Beck-Peccoz P, Lania A, Beckers A, Chatterjee K, Wemeau JL. 2013 2013 European thyroid association guidelines for the diagnosis and treatment of thyrotropin-secreting pituitary tumors. Eur Thyroid J.2(2):76-82. doi:10.1159/000351007.

9. Wang L, Liang H, Deng C, Yu Q, Gong F, Feng F et al. 2022 Functioning gonadotroph adenomas in premenopausal women: clinical and molecular characterization and review of the literature. Pituitary.25(3):454-67. doi:10.1007/s11102-021-01205-9.

10. Fleseriu M, Auchus R, Bancos I, Ben-Shlomo A, Bertherat J, Biermasz NR et al. 2021 Consensus on diagnosis and management of Cushing's disease: a guideline update. Lancet Diabetes Endocrinol.9(12):847-75. doi:10.1016/S2213-8587(21)00235-7.

11. Fan JG, Jia JD, Li YM, Wang BY, Lu LG, Shi JP et al. 2011 Guidelines for the diagnosis and management of nonalcoholic fatty liver disease: update 2010: (published in Chinese on Chinese Journal of Hepatology 2010; 18:163-166). J Dig Dis.12(1):38-44. doi:10.1111/j.1751-2980.2010.00476.x.
